# Real-World Outcomes of Different Types of Treatment for Diabetic Macular Edema Before and After Approval of Anti-Vascular Endothelium Growth Factor Agents

**DOI:** 10.3390/jcm13237336

**Published:** 2024-12-02

**Authors:** Masahiko Sugimoto, Shinichiro Chujo, Kumiko Kato, Masahiko Shimura, Shigehiko Kitano, Sentaro Kusuhara, Hiroto Terasaki, Mineo Kondo, Writing Committee of Japan-Clinical Retina Study Group (J-CREST)

**Affiliations:** 1 Department of Ophthalmology and Visual Science, Faculty of Medicine, Yamagata University, 2-2-2, Iida-nishi, Yamagata 990-9585, Yamagata, Japan; 2 Department of Ophthalmology, Mie University Graduate School of Medicine, Tsu 514-8507, Mie, Japan; shin21@med.mie-u.ac.jp (S.C.); k-kato@med.mie-u.ac.jp (K.K.); mineo@med.mie-u.ac.jp (M.K.); 3 Department of Ophthalmology, Tokyo Medical University Hachioji Medical Center, Hachioji 193-0998, Tokyo, Japan; masahiko@v101.vaio.ne.jp; 4 Department of Ophthalmology, Diabetes Center, Tokyo Women’s Medical University, Tokyo 162-8666, Tokyo, Japan; ge2s-ktn@asahi-net.or.jp; 5 Division of Ophthalmology, Department of Surgery, Kobe University Graduate School of Medicine, Kobe 650-0017, Hyogo, Japan; kusu@med.kobe-u.ac.jp; 6 Department of Ophthalmology, Kagoshima University Graduate School of Medical and Dental Sciences, Kagoshima 890-8544, Kagoshima, Japan; hirototerasaki112@gmail.com

**Keywords:** anti-vascular endothelial growth factor therapy, diabetic macular edema, pars plana vitrectomy, sub-tenon injection of triamcinolone acetonide

## Abstract

**Background/Objectives**: The object of this study was to determine the outcomes of treatments other than anti-vascular endothelial growth factor (VEGF) therapy for diabetic macular edema (DME) before and after the approval of anti-VEGF therapy in Japan. **Methods**: This was a retrospective study registered in the database of the Survey of Treatment for DME (STREAT-DME). A total of 1683 patients treated from 2010 to 2017 were included. The patients were divided into two groups: (1) a pre-group, treated before the approval of anti-VEGF agents (2010–2013, *n* = 771), and (2) a post-group (2014–2017, *n* = 912). Each group was further categorized based on best-corrected visual acuity (BCVA): (i) improved from poor (>0.3 logMAR units) to good (≤0.3 logMAR units) or (ii) decreased from good to poor. **Results**: In the pre-group, 18.5% of patients improved from poor to good BCVA out of the total patient population (*p* < 0.0001), along with 17.3% out of those administered anti-VEGF therapy (*p* = 0.139), 20.5% of those administered a sub-tenon injection of triamcinolone acetonide (STTA, *p* = 0.02), 17.7% (*p* = 0.20) of those administered photocoagulation, and 14.2% of those who underwent pars plana vitrectomy (PPV, *p* = 0.0001). In the post-group, 21.8% had improved BCVA out of the total patient population (*p* < 0.0001), along with 27.2% of those undergoing anti-VEGF therapy (*p* < 0.0001), 16.7% of those administered STTA (*p* < 0.0001), and 27.2% of those who underwent PPV (*p* < 0.0001). **Conclusions**: STTA and PPV are effective to a certain extent, even after the approval of anti-VEGF agents.

## 1. Introduction

Hyperglycemia due to diabetes mellitus (DM) often leads to vascular abnormalities, including angiogenesis, inflammation, hypoxia, and hemodynamic changes that result in the breakdown of the blood–retinal barrier and an accumulation of fluid. Diabetic macular edema (DME) is a sight-threatening complication of diabetic retinopathy, and more than 20 million individuals worldwide suffer from it [1].

Various treatments have been developed to treat DME, including (1) photocoagulation (PC) [2]; (2) steroids, such as an intravitreal injection of triamcinolone acetonide (IVTA) [3] or a sub-tenon injection of triamcinolone acetonide (STTA) and sustained-release intravitreal steroids such as Ozurdex^®^ (dexamethasone, Allergan Inc., Irvine, CA, USA) and Iluvien^®^ (fluocinolone acetonide; Alimera Science, Alpharetta, GA, USA) [4,5]; and (3) pars plana vitrectomy (PPV) [6].

The management of DME has markedly changed over the past decade. In fact, in the 2010s, large clinical trials demonstrated that anti-vascular endothelial growth factor (VEGF) therapy improved visual acuity (VA) significantly more than PC or IVTA [7,8]. At present, anti-VEGF therapy has replaced PC as the first-line treatment for DME [9]. However, other treatments, such as PC, IVTA, STTA, and PPV, are still used with careful consideration of a patient’s background in real-world clinical practice.

We recently developed the Survey of Treatment for DME (STREAT-DME) study in 2020 [10,11]. This was a multicenter registry of patients with DME in Japan. Two earlier reports in 2010 and 2017 presented the outcomes and progression of DME following various types of treatments and transitions. The results showed that the trend in treatments used changed dramatically around 2013 when anti-VEGF agents were approved in Japan. Although anti-VEGF therapy has been shown to be very effective, it poses some challenges in real-world clinical practice, such as the need for multiple injections for maintenance, the accompanying economic or time burden, and the possibility of systemic side effects.

Although anti-VEGF therapy has become the first-line treatment, it does not negate the effectiveness of other treatments. Therefore, the purpose of this study was to evaluate the efficacy of treatments other than anti-VEGF therapy for DME before and after the approval of anti-VEGF agents.

## 2. Materials and Methods

The STREAT-DME database contains the medical records of a demographically and geographically diverse patient population obtained from 41 retina specialists at 27 ophthalmological institutions in Japan [10,11]. This retrospective observational study included all eligible patients who received a diagnosis of center-involving DME with initial treatment between January 2010 and December 2015 (follow-up range: 22–26 months). Registered data were collected from 2010 to 2017. Among a total of 2049 eyes with treatment-naïve DME, patients who received a single treatment were included. In short, those who underwent a treatment pattern of monotherapy were included, and those who underwent a treatment pattern of combination or switch therapy were excluded.

DME was diagnosed at each institution, and the timing of treatment was decided by the attending physician. The treatment for DME classified as anti-VEGF therapy consisted of 1.25 mg/0.05 mL of intravitreal bevacizumab (Avastin^TM^; Genentech, San Francisco, CA, USA), 0.5 mg/0.05 mL of intravitreal ranibizumab (Lucentis^TM^; Genentech), or 2.0 mg/0.05 mL of intravitreal aflibercept (Eylea^TM^; Regeneron Pharmaceuticals, Tarrytown, NY, USA). The other treatment options included 4 mg/0.1 mL of IVTA or 20 mg/0.5 mL of STTA, PC of the macular region, or PPV. If cataract surgery was performed or PC outside the macular region was performed to prevent retinal ischemia during the 2-year period, this was also recorded in case it influenced the visual prognosis. 

### 2.1. Clinical Evaluations

The best-corrected visual acuity (BCVA) was measured using a decimal visual acuity chart on the initial and final visits during the 2-year period. Interventions for each eye were also recorded. To facilitate data analyses, the decimal BCVAs were converted to the logarithm of the minimal angle of resolution (logMAR) units. Because the goal of treating DME was to maintain a useful BCVA, the percentage of eyes with a final BCVA ≥ 0.3 logMAR units (≥20/40 of ETDRS equivalent letter scores) was also calculated. A value of 0.3 logMAR units was selected because it represents socially useful vision and is defined as a ‘good’ BCVA. In contrast, a BCVA < 0.3 logMAR units was defined as poor BCVA.

The patients were divided into two groups: (1) the pre-group, who started DME treatment before the approval of the anti-VEGF agents, from 2010 to 2013, and (2) the post-group, who started DME treatment after the approval of anti-VEGF agents, from 2014 to 2017. In addition, both groups were further categorized based on their BCVA as follows: (i) “Improvement”, having a BCVA that improved from poor (>0.3 logMAR units) to good (≤0.3 logMAR units), or (ii) “Deterioration”, having a BCVA that decreased from good (≤0.3 logMAR units) to poor (>0.3 logMAR units).

### 2.2. Statistical Analyses

McNemar’s test was used to determine the significance of differences among the skewed variables. The chi-squared test was used to determine the significance of differences among the groups. Statistical significance was defined as a two-tailed *p*-value < 0.05. Analyses were performed using SAS V.9.4 TS1M5 (SAS Institute, Cary, NC, USA) and were carried out at an independent biostatistics data center (STATZ Institute, Tokyo, Japan).

## 3. Results

### 3.1. Demographics of Patients (Table 1)

At the time of the statistical analyses, there were 2166 eyes in the STREAT-DME database. Based on the inclusion criteria, 1683 treatment-naïve eyes were included in this study. The pre-group consisted of 771 eyes, and the post-group consisted of 912 eyes. The demographics of the patients in each group are presented in Table 1.

**Table 1:** Demographics of patients.

	2010–2013	2014–2017	Total
	(Pre, Eyes)	(Post, Eyes)	(Eyes)
Anti-VEGF	133	294	427
IVTA	24	37	61
STTA	239	269	508
PC	198	187	385
PPV	177	125	302
Total	771	912	1683

Anti-VEGF, anti-vascular endothelial growth factor therapy; IVTA, intravitreal injection of triamcinolone acetonide; PC, photocoagulation; PPV, pars plana vitrectomy; STTA, sub-tenon injection of triamcinolone acetonide. Pre-group: patients who received treatment before the approval of anti-VEGF agents (2010–2013); post-group: patients who received treatment after the approval of anti-VEGF agents (2014–2017).

### 3.2. BCVA Improvement for Each Type of Treatment Among the Pre- and Post-VEGF Groups

The comparisons between the pre- and post-groups for each type of treatment are shown in Table 2. For the total patient population, significant improvements in BCVA from poor to good were observed in all of the pre-group patients (18.5%, *p* < 0.0001; McNemar’s test) and all of the post-group patients (21.8%, *p* < 0.0001). For the patients who received anti-VEGF therapy, BCVA improvement from poor to good was significantly higher in the post-group (27.2%, *p* < 0.0001) compared to the pre-group (21.8%, *p* = 0.139). BCVA improvement from poor to good was significantly more prevalent in both the pre- and post-groups among the patients who received STTA (20.5% in the pre-group [*p* = 0.02] and 16.7% in the post-group [*p* = 0.001]) or PPV treatment (19.2% in the pre-group [*p* = 0.0001] and 27.2% in the post-group [*p* = 0.001]). BCVA improvement from poor to good was not significant among the patients who received IVTA or PC treatment in both the pre- and post-groups.

### 3.3. Relative Ratio of Patients with BCVA Improvement or Deterioration for Each Treatment

A comparison of the relative ratio of patients with BCVA gain (poor to good BCVA) or loss (good to poor BCVA) for each treatment between the pre- and post- groups is shown in Table 3. For the total patient population, the percentage of patients with an improvement in BCVA was significantly higher (21.8%, *n* = 199) in the post-group compared to the pre-group (18.5%, *n* = 143; *p* = 0.015, chi-squared test). The percentage of patients with a deterioration in BCVA was not significantly different between the pre-group (10.6%, *n* = 82) and the post-group (8.3%, *n* = 76).

The percentage of patients with an improvement in BCVA was significantly higher in the post-group (27.2%, *n* = 80) compared to the pre-group (17.3%, *n* = 23) among the patients who received anti-VEGF therapy (*p* = 0.013). Among the patients who received anti-VEGF therapy, the percentage of patients with a deterioration in BCVA was not significantly different between the pre-group (10.5%, *n* = 14) and the post-group (7.5%, *n* = 22).

Among the patients who received IVTA, STTA, PC, or PPV treatments, there were no significant differences in the patients with an improvement in BCVA between the pre- and post-groups. There was also no significant difference in the percentage of patients with a deterioration in BCVA among the patients who received these treatments.

## 4. Discussion

Anti-VEGF therapy has overtaken other treatment options to become the first-line treatment for DME. Because the approval of anti-VEGF agents enables us to use agents much more freely and not only for patients with advanced-stage disease, the relative ratio of patients with BCVA improvement has increased from 17.3% to 27.2% for anti-VEGF-treated groups. However, because anti-VEGF therapy is not necessarily effective for all patients, some patients should be treated with other therapies. Because we found that the percentage of BCVA improvement for STTA and PPV remained high even after the approval of anti-VEGF agents, they are still effective treatment options in a certain percentage of patients. But here, we could not find any significant difference among the patients treated with IVTA due to the small number of them.

Previous studies assessing DME treatment outcomes have found that anti-VEGF therapy has accounted for an increasing proportion of treatments over time, and the frequency of other treatments is dwindling [12,13]. However, these studies were conducted using an insurance database; thus, treatment outcomes could not be analyzed. Given that our results are based on patient registries from real-world practice, our analyses not only included the types of treatments but also their outcomes. Here, we found that treatments other than anti-VEGF therapy are still effective for DME, even in the current era of anti-VEGF therapy.

A questionnaire survey of vitreoretinal specialists in Japan found that the financial cost and frequency of injections were important barriers to anti-VEGF therapy, and patients with vascular infarction diseases were discouraged from receiving anti-VEGF therapy [14]. Furthermore, financial and other characteristics have also been reported to be important for the decision to administer the treatment [15]. Avery et al. reported an increased risk of death among DME patients with higher exposure to anti-VEGF agents from registration-level randomized controlled trial (RCT) data [16]. On the other hand, various other RCTs and meta-analyses have reported no such risk [17,18,19]. Nevertheless, there are warnings on the medication instruction form about the administration of anti-VEGF agents to patients with underlying conditions such as thromboembolic events (Lucentis^TM^ prescribing information from Genentec, https://www.gene.com/patients/medicines/lucentis, accessed on 15 November 2024). Therefore, it is possible that administration for these patients is intentionally avoided [14], and treatments other than anti-VEGF therapy in real-world clinical practice are chosen as the choice of treatment, as shown in our study.

After the introduction of anti-VEGF therapy into clinical practice for DME, IVTA lost its position as the first-line therapy. Although many reports have shown the usefulness of STTA for DME [20,21,22], STTA has never been the first-line choice of treatment anywhere in the world, including Japan. This is due to a lack of consensus on the efficacy and side effects associated with steroids. The DRCR.net Protocol-E reported that no important clinical effects were observed after STTA compared to focal PC. However, it is still unclear whether STTA is effective or not as they recruited patients with better BCVA (≥20/40) compared to the previous small-group study [23]. Thus, the usefulness of STTA should not be ignored, as our results confirm the effectiveness of STTA even in the era of anti-VEGF therapy within a certain percentage of patients.

PPV is also known to be effective in resolving DME with abnormal vitreoretinal interfaces, such as traction associated with a thickened and taut pre-macular posterior hyaloid [6]. In fact, various studies have shown that vitrectomy is effective for DME [24,25,26,27]. For example, the European Vitreo-Retinal Society conducted a non-randomized multicenter study of 2603 patients with macular edema, including 870 patients with DME. The study reported that PPV led to better visual improvement than the other treatments [28]. However, for the DRCR.net. Protocol-D, it was reported that even though BCVA improved in some patients, BCVA worsened in other patients, leading to the conclusion that PPV requires further investigation as a DME treatment option [29]. Furthermore, a meta-analysis of 11 randomized controlled studies reported that there was little evidence to support the idea that PPV is effective for DME in the absence of the epiretinal membrane or vitreomacular traction [30]. Thus, PPV could be effective if appropriate case selection (i.e., DME due to vitreomacular traction) is performed, regardless of the approval of anti-VEGF agents.

There are some limitations to our study. First, because this study is based on data from 2017, the actual real-world situation may have changed. To this end, we are currently constructing a new patient registry to collect up-to-date data that we plan to publish as a new dataset in the future. Second, the timing and choice of each treatment were decided by each physician based on the background of individual cases, such as a patient’s general condition or the initial appearance of the vitreoretinal interface. Future studies must take these problems into account. Third, this study included a total of 1683 eyes from 27 institutions, which is not enough for a real-world study. This is because we excluded patients who received combination or switch therapy from a total of 2049 patients. We need a much larger number of patients for further investigation. Finally, we defined the cut-off value for BCVA improvement as 0.3 log MAR in this study. Based on this definition, BCVA improvement is fundamentally affected by the baseline visual acuity because of the possibility that the same two-line improvement may be classified into different outcome groups. A previous study stated that the percentage of eyes improved by 15 letters [10]. For the accurate evaluation of treatment efficacy, we also need to compare results from different cut-off values with our result. Since this survey was developed in 2017, we need further investigation for the decade following the approval of anti-VEGF therapy.

## 5. Conclusions

In conclusion, a subgroup analysis of the STREAT-DME registry showed that STTA and PPV are still effective treatments, even in the era of anti-VEGF treatment. Although the approval of anti-VEGF agents for DME has expanded, treatments other than anti-VEGF therapy remain viable options and may contribute to favorable outcomes for patients with DME.

## Figures and Tables

**Table 2:** BCVA improvement for each type of treatment in the pre- and post-groups.

	2010–2013	*p*-Value	2014–2017	*p*-Value
	(Pre)		(Post)	
Anti-VEGF	17.3 (%)	0.139	27.2 (%)	<0.0001 **
	134 (*n*)		80 (*n*)	
IVTA	8.3	0.44	16.2	0.45
	2		8	
STTA	20.5	0.02 *	16.7	0.001 **
	49		45	
PC	17.7	0.20	18.2	0.08
	35		34	
PPV	19.2	0.0001 **	27.2	0.001 **
	34		34	
Total	18.5	<0.0001 **	21.8	<0.0001 **
	254		201	

Anti-VEGF, anti-vascular endothelial growth factor therapy; IVTA, intravitreal injection of triamcinolone acetonide; PC, photocoagulation; PPV, pars plana vitrectomy; STTA, sub-tenon injection of triamcinolone acetonide. Pre-group: patients who received treatment before the approval of anti-VEGF agents (2010–2013); post-group: patients who received treatment after the approval of anti-VEGF agents (2014–2017). * *p* < 0.05, ** *p* < 0.01, McNemar test.

**Table 3:** Relative ratio of patients with BCVA improvement or deterioration for each treatment.

		2010–2013	2014–2017	*p*-Value
		(Pre, %)	(Post, %)	(Pre vs. Post)
Anti-VEGF	(Poor to Good)	17.3	27.2	0.013 *
	(Good to Poor)	10.5	7.3	n.s.
IVTA	(Poor to Good)	8.3	16.2	n.s.
	(Good to Poor)	10.8	11.9	n.s.
STTA	(Poor to Good)	20.5	16.7	n.s.
	(Good to Poor)	10.4	11.8	n.s.
PC	(Poor to Good)	17.7	18.2	n.s.
	(Good to Poor)	12.4	12.6	n.s.
PPV	(Poor to Good)	19.2	27.2	n.s.
	(Good to Poor)	7.3	9.2	n.s.
Entire	(Poor to Good)	18.5	21.8	0.015 *
	(Good to Poor)	10.6	8.3	n.s

Anti-VEGF, anti-vascular endothelial growth factor therapy; IVTA, intravitreal injection of triamcinolone acetonide; PC, photocoagulation; PPV, pars plana vitrectomy; STTA, sub-tenon injection of triamcinolone acetonide. Pre-group (pre): patients who received treatment before the approval of anti-VEGF agents (2010–2013); post-group (post): patients who received treatment after the approval of anti-VEGF agents (2014–2017). BCVA: best-corrected visual acuity. * *p* < 0.05; n.s., not significant; chi-squared test.

## Data Availability

The datasets used in the current study are available from the corresponding author (Masahiko Sugimoto, Department of Ophthalmology and Visual Science, Faculty of Medicine, Yamagata University) upon request (email: sugmochi92@gmail.com). The study protocol was registered with the University Hospital Medical Information Network individual case data repository (UMIN000023160, shown in https://center6.umin.ac.jp/cgi-open-bin/ctr/ctr_view.cgi?recptno=R000026698, accessed on 15 November 2024).
